# Development of a self-management intervention for employees with complaints of the arm, neck and/or shoulder (CANS): a focus group study with experts

**DOI:** 10.1186/s12995-015-0051-z

**Published:** 2015-02-27

**Authors:** Nathan Hutting, Josephine A Engels, J Bart Staal, Yvonne F Heerkens, Maria W G Nijhuis-van der Sanden

**Affiliations:** Radboud Institute for Health Sciences, IQ Healthcare, Radboud University Medical Center, Nijmegen, The Netherlands; Research Group Occupation and Health, HAN University of Applied Sciences, PO Box 6960, Nijmegen, 6503 GL The Netherlands

**Keywords:** Complaints of the arm, Neck and/or shoulder, CANS, Work-related upper extremity disorders, WRUED, Focus group, Self-management

## Abstract

**Background:**

Many people suffer from complaints of the arm, neck and/or shoulder (CANS). The complaints are persistent and there is a need for intervention programs for those with longstanding CANS. Studies suggest that a behavioural change is needed in employees with CANS. A self-management program with an add-on eHealth module might be an effective option to achieve the behavioural change needed to manage the complaints in employees with CANS. The aim of this study was to determine the content and strategies of the intervention and to gain insight into possible barriers and facilitators for implementation. Therefore, we examined the views of experts on the problems and characteristics associated with employees with CANS as well as their opinion on a self-management program consisting of self-management sessions and an eHealth module.

**Methods:**

A qualitative study was performed consisting of three focus groups involving a total of 17 experts (with experience with CANS, self-management and/or eHealth interventions). Experts were asked their opinion about the content and requirements of a self-management program for employees with CANS, including an eHealth module. Data were analysed using qualitative data analysis. After coding, the emergent themes were used to organise the data into main categories, expressing the ideas and opinions of experts on CANS, self-management and/or eHealth interventions.

**Results:**

The experts pointed out that the intervention should focus on increasing employees’ self-efficacy and empowerment, and address topics related to the possible risk factors for CANS, symptoms, work environment, social environment and personal factors. The eHealth module should be self-explanatory and attractive, and the information provided should be brief, clear and concise.

**Conclusions:**

Experts appeared to see a role for a self-management program for employees with CANS. They indicated that the combination of group sessions and eHealth can work well. Experts provided valuable information with regard to the content of the self-management intervention and the design of the eHealth module.

## Background

Many people suffer from complaints of the arm, neck and/or shoulder (CANS). Reported point prevalence for this disorder ranges from 1.6–53% and the 12-month prevalence from 2.3–41% depending on the setting, definition, and classification used [1-3]. In various working populations the reported 12-month prevalence ranges from 22–40% [3].

Although the exact aetiology of nonspecific CANS is unknown, it is reported to be of multifactorial origin in which work-related factors may play a major role [4-7]. Physical characteristics (i.e. awkward working posture, repetitive work), psychosocial characteristics (i.e. lack of social support from colleagues or supervisor), personal factors (i.e. an ineffective approach to stress management) of the individual worker, as well as characteristics of their work environment (i.e. high job demands, lack of control), contribute to the development and persistence of complaints [4-13]. The importance of each factor, and its individual contribution to the risk of provoking symptoms, varies among individuals and work environments [14].

CANS may cause significant work problems, including absenteeism (sickness absence), presenteeism (decreased work productivity) and, ultimately, job loss [15,16]. A recent focus group study showed that employees with CANS have to deal with their complaints in their daily life and at work [17]. That study also showed that participants are not fully aware of the possibilities to influence their complaints, or of their own role in coping with their complaints. Participants generally suffer from pain, are often approaching their individual limits, and fatigue has a major impact on their life; in addition, they also have to deal with hindering physical and social environmental factors, such as misunderstanding from others [17]. Employees with CANS are often confronted with a wide range of problems. Most have taken many steps in an attempt to reduce their complaints, which can vary from workplace adaptations to different types of (physical) therapies [17].

About 19% of the patients report chronic complaints of which 58% report the use of healthcare, such as care given by the general practitioner, medical specialist and physical therapist [1]. Thus, there seems to be a need for intervention programs for people with longstanding CANS [17-19]. Therefore, information on the experiences and needs of employees with CANS can be valuable in developing such interventions. To determine the content of the intervention, and to guarantee that strategies will be adopted and implemented, the perspective of the healthcare professional should also be taken into account, because they refer clients to these programs or give the care themselves. Experts on care for those with CANS can probably provide valuable information that can be used in the development of intervention programs for employees with CANS.

Self-management is an approach increasingly used in chronic disease care to improve self-efficacy and a healthy lifestyle [20]. Self-management interventions focus primarily on encouraging patients to be involved with and in control of their own treatment, as well as improving their understanding of how their condition and treatment affect their lives [21]. Self-management often includes preparing people to manage their health behaviours on a day-to-day basis, participating in treatment or education designed to attain specific results, practicing tasks, and developing attitudes that reduce the emotional or physical impact of illness, with or without assistance from clinicians [22]. There is inconsistent evidence for the effects of self-management programs for patients with chronic musculoskeletal pain [23-25] and there is some evidence that group-delivered short programs (< 8 weeks) with a healthcare professional involved have the best potential [23].

A promising medium for facilitating patient empowerment is the Internet [26]. Many home-based disease-management programs have been developed to improve the health of patients [27]. eHealth interventions have become popular in number and reach [28]. A recent systematic review indicates that web-based interactive interventions have a beneficial effect on patient empowerment and/or physical activity in patients with various chronic conditions [26].

Unfortunately, web-based interventions also have some possible disadvantages. For example, it is common for users who experience difficulties with the program to discontinue program use or drop out of a study before completion [29,30]. Moreover, for the specific group of participants with CANS, who often work with computers at work, more prolonged computer use (by following an eHealth program) could worsen their physical problems [31]. Also, eHealth alone limits the (often very supportive) personal contacts between participants. Therefore, a combination of a self-management program with an add-on eHealth module could be an effective option to achieve behavioural change in the management of complaints in employees with CANS, especially in those suffering from longstanding complaints. CANS has a multifactorial origin and symptoms are diverse; by adding an eHealth module, information can be provided in a more tailored way (in which participants can make their own choices) [17]. In this way, the time during the meetings can be used more effectively, whilst relevant information is available at every moment due to the eHealth module. Our research group plans to adapt the self-management program developed by Detaille et al. [32,33] following the process of intervention mapping [34,35] and add an eHealth module for use in employees with CANS persisting for ≥ 12 weeks.

The aim of the present explorative study is to determine the content and strategies of the self-management program and eHealth module and to gain insight into possible hindrances and facilitators for implementation. Therefore, this study evaluates the experiences and opinions of experts in the field of CANS, self-management and/or eHealth, regarding the problems and characteristics of employees with CANS, as a step towards developing a self-management program consisting of self-management sessions and an eHealth module. Using this information, the existing self-management program developed by Detaille et al. [32,33] can be adapted and designed to fit the needs of the target population in order to make it easier for them to achieve healthy behaviours and management of their symptoms.

## Methods

### Study design

In March 2012, three focus groups with experts in the field of CANS, self-management and/or eHealth were held. Two focus groups were held at the HAN University of Applied Sciences, Nijmegen, and one focus group was held at a hotel in Utrecht (both in the Netherlands). The Medical Ethical Committee at Radboud university medical center declared (registration number 2013/316) that the study does not fall within the Dutch law on ‘Medical Research involving Human Subjects’ (the WMO) and that therefore, no approval is required from a medical ethic committee. The research protocol fulfilled the criteria of the Declaration of Helsinki on Ethical Principles for Medical Research Involving Human Subjects.

We used focus groups to investigate the broad range of ideas that experts had about CANS, self-management and eHealth. Focus groups can uncover factors that influence opinions, behaviour or motivation [36] and provide an interactive environment in which ideas can emerge from the group [36]. A group possesses the capacity to become more than the sum of its parts and to exhibit a synergy that individuals alone do not possess [36]. Therefore, focus groups were considered the most suitable tool to address the aim of this study.

### Participants

Between January and March 2012, a purposive sampling technique was used to identify potential participants. Experts (defined as persons with a high degree of practice skills or knowledge, i.e. relevant postgraduate professional qualifications, a PhD, and/or experienced clinicians) in the field of CANS, self-management and/or eHealth interventions were identified by the authors, contacted by email or telephone, and asked to participate in the study. The different areas of expertise represented by the experts were distributed over the three focus groups, thereby ensuring that all areas of expertise were represented in all focus groups. Each participant was informed that participation was voluntary and that data would be used anonymously. All participants were asked to fill out a short questionnaire (demographics) prior to the start of the focus group. All participants agreed to audio-recording of the sessions. All participants received a gift of 75 euro for their participation.

A total of 17 experts, divided in three groups of five or six participants each, participated in this focus group study; of these, 12 (70.6%) were female and five (29.4%) were male. The mean age of the participants was 45.3 (range 28–60) years. The demographic profile of the participating experts is presented in Table 1. Experts worked in various professions. All participants had postgraduate qualifications in the field of CANS, eHealth and/or self-management. Five experts (29.4%) had a higher professional education and 12 (70.6%) had an academic higher education. Of the participants, 64.7% was an expert on CANS, 64.7% an expert on self-management, and 58.8% an expert on eHealth (some experts had more than one kind of expertise).

**Table 1:** **Demographic profile of the participating experts**

**Expert number**	**Gender**	**Age (years)**	**Group**	**Education level**	**Education**	**PhD**	**Profession**	**Expertise**		
								**CANS**	**SM**	**EH**
1	Female	60	1	AHC	Psychology	Yes	Researcher	No	Yes	No
2	Female	44	1	HPE	Physiotherapy, occupational health physiotherapy	No	Physiotherapist	Yes	Yes	Yes
3	Male	57	1	AHC	Medicine	No	Occupational health physician	Yes	Yes	No
4	Female	31	1	AHC	Occupational therapy, health sciences	No	Occupational therapist, lecturer	Yes	No	No
5	Female	50	1	HPE	Work and Organization	No	Work and Organization expert	Yes	No	No
6	Female	45	1	HPE	Physiotherapy, occupational health physiotherapy	No	(Occupational health) physiotherapist	Yes	Yes	No
7	Female	53	2	HPE	Management	No	Health and Safety Coordinator	Yes	No	No
8	Female	47	2	AHC	Speech therapy, speech and language pathology	PhD.c	Researcher	No	Yes	Yes
9	Female	30	2	AHC	Psychology	PhD.c	Researcher	No	Yes	Yes
10	Male	47	2	AHC	Occupational therapy	PhD.c	Lecturer, researcher	Yes	Yes	No
11	Female	47	2	HPE	Occupational therapy	No	Occupational therapist	Yes	Yes	Yes
12	Female	42	3	AHC	Health sciences	Yes	Researcher	Yes	No	Yes
13	Male	33	3	AHC	Movement sciences, epidemiology	Yes	Product manager eHealth	No	No	Yes
14	Male	56	3	AHC	Physiotherapy, occupational health physiotherapy	No	(Occupational health) physiotherapist	Yes	Yes	Yes
15	Female	42	3	AHC	Health sciences	Yes	Researcher	No	Yes	Yes
16	Female	58	3	AHC	Sociology	Yes	Researcher, development	No	Yes	Yes
17	Male	28	3	AHC	Physiotherapy, health sciences	PhD.c	Researcher	Yes	No	Yes

HPE = Higher professional education, AHC = Academic higher education, CANS = Complaints of arm, neck, and/or shoulder, SM = Self-management, EH = eHealth, PhD = Doctor of Philosophy, PhD.c = Doctor of Philosophy candidate.

### Focus groups

Following the recommendations of Krueger and Casey [36] a semi-structured interview guide with open-ended questions was developed (Appendix) by the authors. The interview guide was based on the expertise of our research group (NH, JE, BS, YH, MN, and SD). The expertises included: guideline development, self-management, work-related disorders, and clinical experience with CANS. The interview guide was pilot-tested in the first focus group and, because no modifications were necessary, we used the same interview guide in all three focus groups. The group members were asked for their opinion and experiences on CANS and/or self-management, including an eHealth module. Moreover, they were asked for their ideas on the content of the self-management intervention, and the requirements to be fulfilled by the eHealth module and the self-management sessions. Moreover, possible barriers and facilitators were explored. The participants had no knowledge about the results of the focus groups held earlier with employees with CANS, described in an earlier article [17] and no other information was provided. Each focus group was moderated by the first author (NH) using a standardized script. All focus groups were audio-recorded and notes were taken by an assistant (LD). In each meeting the pre-developed interview guide was followed. The moderator made sure that every participant was involved in the discussion. The moderator actively generated interaction and discussion between participants. Each of the three sessions lasted about 120 min. After each session, the moderator and the assistant discussed the group dynamics and made a summary of the most striking results [36].

### Data analysis

The audio-recordings were transcribed by an assistant (LD). The draft version of the Results section was sent to all participants and they were asked to screen the text for misinterpretations and to make additions if necessary. If the participants did not respond to the first email within 10 days, one reminder was sent to them by email. The first author (NH), who was trained in qualitative research methods, performed the data analysis.

After reading each transcript multiple times, the transcripts were analysed using qualitative data analysis with an open-coding system [37]. New codes were added when considered necessary. After that the codes were sorted into themes based on how the different codes are related and linked [37]. Then the emergent themes were used to organise the data into main categories [37], expressing the ideas and opinions of experts on CANS, self-management and/or eHealth interventions. Moreover, the relationship between the categories was explored [37].

The Atlas.ti (version 7.082) program was used for analysis. During data analysis, the emerging themes were discussed in the research group. Moreover, by reading all the transcripts, the research group checked that no main categories were missed. The supporting quotes related to each theme were discussed by the research group.

## Results

**Figure 1:**
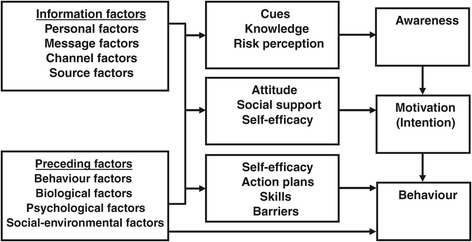
**The I-Change model (2.0) [** 38 **].**

With regard to the development of the intervention the experts indicated, for example, that insight into the complaints and self-awareness and knowledge about the complaints (e.g. about risk factors) are important. They also stated that self-management starts as a personal problem of the employee and that it is important that the employee him/herself is in control. The attitude of employees towards their complaints and possible social support was also considered important. During data analyses, it appeared that these categories emerging from the data showed similarities with the I-Change model (2.0) (Figure 1), which consists of three phases of behavioural change [38]. Therefore, the derived categories with regard to the content of the intervention were clustered according to the three phases (Awareness, Motivation and Behaviour) of the I-Change model (2.0) [38]. Moreover, experts gave their opinions on the combination of self-management sessions and an additional eHealth module, and the conditions and requirements concerning the eHealth module.

All data and information presented here are the opinions and statements of the experts.

### Awareness

#### Insight into the complaints

Experts in the focus groups stated that employees with CANS have to work on identifying factors regarding the onset and persistence of their complaints. Experts considered it important that, at the start of the intervention, participants evaluate their individual problem areas, gain insight into their complaints, and develop self-awareness. It was mentioned that identifying risk factors and contextual factors can start in the self-management sessions, because here people feel most at ease and reassured. In addition, the eHealth module can be helpful because it can give additional explanations and background information. According to the experts, it also seems advisable to involve an expert on CANS in the program, to provide information and to answer company-specific questions of the participants.

Some experts indicated that for successful participation in a self-management program participants should have a certain cognitive level and must have a learning capacity. Participants in the program must have the ability to perform self-reflection and to look critically at their work environment, colleagues and at themselves. For awareness and self-reflection a considerable amount of information must be available and appropriate tools must be provided during the intervention. People need to examine their own problems and address them individually. Incorporating a screening tool or test to gain insight into their own situation and contributing factors is also advisable.

#### Putting priority on the health problem

Listening to the body was considered important. One expert said that if workers have complaints for ≥ 12 weeks, then they have not listened properly to their body. Self-awareness was indicated as one of the most important items; in addition, employees must be able to manage their own workload and complaints. One expert said:

*It’s striking that most of the employees who I see are under a lot of pressure at work and take almost no breaks… and then they also have difficulty in being able to self-manage. Because they think that they have to finish their work, there is no time for a break. Then you come into a type of conflict situation.* (Expert 4)

Experts stated that many employees have a high workload and take almost no breaks, leading to a type of conflict, because they feel they cannot take the time to take these breaks. Therefore, it is difficult to manage their own health problems and the workload. Especially for this group of workers, awareness and behavioural change were considered important. Complaints do not always go away, but a self-management program could offer support to these employees. One expert stated:

*At the time that someone personally achieves insight into the causality of the story and can thereby also take control into his/her own hands, then you retain someone in the work process. (…) As such a person is then busy with self-correction. (…) I consider this to be the most meaningful activity that you (…) can provide.* (Expert 3)

This latter view was widely shared. It was stated that employees with CANS must put priority on their own health problem. Experts stated that it is important to be aware of the relationship between complaints and their causes and that individuals realize that they need to change their behaviour.

#### Knowledge about the complaints

Experts agreed that providing relevant knowledge can be a part of a self-management intervention. This can also create cues that prompt people to become aware. Awareness with regard to possible risk factors and how participants can influence these risk factors themselves, can be a cue to take action. In general, experts believed it is important that employees with CANS get information about their complaints, e.g. regarding symptoms such as pain, tingling, muscle spasms and loss of coordination. Moreover, most experts found it important that the intervention deals with possible risk factors related to the complaints. This also facilitates risk perception of the employees. The diversity of these topics is often not known and all these topics should be addressed. For employees with CANS, clarity of information is important. Experts agreed that the risk factors related to CANS are multifactorial and that causes can vary from person to person:

*Quite often the causes simply arise from the relationship with the boss or employer… but the cause can also arise from a large number of other things. That your office material or equipment is not right, or your monitor is not good. Or just because you don’t feel comfortable in the group, or you’re having family problems.* (Expert 15)

Experts found it important to address the reasons why employees can be overloaded. They also found it important to address possible risk factors related to the persistence of the complaints, which employees are often unaware of. Employees must be aware that the body gives signals of overload. These signals should be a cue to take action. Experts stated that employees often carry on too long and often fail to take action until it is too late.

Experts believed it to be important to discuss the potential risk factors related to CANS, for instance, by giving some general examples and explaining the effects of several risk factors on the onset of complaints. The employee’s behaviour was seen as an important factor related to the onset of symptoms. Some experts suggested possible risk factors that are important to discuss in the intervention: (work) stress, posture, workplace (materials and equipment), work tasks (repetitive tasks, extreme workload, extreme positions of joints), social factors (colleagues, relationship with supervisor), personal circumstances, and lack of physical activity.

In one focus group there was some discussion about the role that work plays as a cause of the onset of symptoms:

*I agree that you have to do something about the pain, I don’t agree that all of these non-specific complaints are caused by work. They are relevant to carrying out the work, they impede the work, and perhaps it’s difficult to recover from these complaints if no accommodation is made in the work environment. But I don’t know if it’s always the cause … but it is work-related.* (Expert 12)

This quote indicates that work is considered a factor in the onset and chronic character of symptoms and that complaints are believed to have at least a relationship with work. However, experts indicated that the actual work itself is not necessarily the cause of the complaints.

Some experts indicated that employees with CANS generally have high demands (on themselves) and are often perfectionists. One expert stated that particular highly educated employees develop CANS and that the content of the work may also play a role:

*It’s also a combination of stress and a high level of pressure at work. I also often see data typists, these people listen to music and are thus inputting things … that’s very repetitive work but these people often have less problems. And what I really have noticed is that these dedicated ITers, who also work on the computer at home for an extra 8 hours, have no complaints at all. It’s often a combination of self-imposed stress and actual stress and repeated movements. Because of the deadlines and self-imposed stress they work through the pain.* (Expert 14)

Some experts also indicated that, in employees with CANS, the problem is less related to the workplace itself than to the behaviour (i.e. experiences and the intensity) of the employee at work. Generally, employees with complaints for ≥ 12 weeks have already tried many different options related to work adaptations. Regarding the causes, one expert stated:

*It more closely resembles a burn-out than an irritation of a tendon or capsule. The intensity at which people work affects the development of complaints much more than the physical conditions of the work environment. After 12 weeks it really is more about the psychological aspects.* (Expert 14)

Taken together, experts stated that it is important to inform employees with CANS about all possible causes and potential risk factors, and to stimulate them to analyse their own situation. Moreover, experts stated that the intervention should focus on psychosocial aspects, especially during the self-management sessions. Moreover, it was stated that working conditions and environmental factors can be discussed in the eHealth module.

### Motivation

#### Motivation for making changes

Experts agreed that self-management begins with the intention to take action; this is a prerequisite for a chance of success. Motivation is an important condition, participants must see the need for a change and the need to have control in their disease management.

*Then it doesn’t matter whether someone is working somewhere for a sheltered workplace or whether that person is a manager at the Shell Corporation. Both will go well, as long as the motivation is present to do something about it.* (Expert 11)

Experts agreed that self-management should start from a personal problem experienced by an employee. This ensures sufficient motivation. One has to recognize the possibilities to make changes. It is very important that people come up with their own solutions, are in control, and feel empowered. In the situation that healthcare professionals are involved, it is important that they support the client, but that the client stays in control and indicates his/her needs. One expert stated:

*Self-management is by definition oriented towards decision-making. Therefore, you need a problem, which means that this person her/himself has to have a problem. Then you can come with (amongst others) some knowledge, or with advice and counselling, that can be instrumental - but self-management starts with a problem that you yourself have.* (Expert 1)

A barrier of self-management is that clients may be (too) passive. Therefore, healthcare professionals must be aware of this and facilitate the client to stay in control. According to most experts, it is important that healthcare professionals involved in self-management also undergo a change themselves.

#### Attitude towards the complaints

Experts stated that employees with CANS must be proactive rather than reactive. One must take action and make changes. People have to think about what they need to make a successful change; it is important that they think in terms of possibilities rather than problems. Concerning ‘positive thinking’ one expert remarked:

*People often adopt the attitude that ‘I can’t do this anymore’ - whereas you have to turn them around to adopt the attitude that ‘I simply have this condition at this moment in time but I can still do other things’. Therefore, they have to actually see the opportunities rather than the limitations.* (Expert 7)

Experts believed that by providing information about CANS and by understanding the course of the disorder, the attitude of employees with CANS can be influenced. For example, in the chronic stage the pain can be present continuously, the course can also be erratic, and it can take a long time before the changes made have an effect. Therefore, to change the attitude towards pain, providing information about pain is considered important; for example, about what (chronic) pain is and what the function of pain is. Employees should be aware of this and understand it. Learning to deal with the pain is important. Attention should also be paid to the emotions that arise with pain, the cognitive aspects surrounding pain, and the use of pain medication. Regarding the experienced pain one expert said:

*It’s quite different when people suffer pain for 12 weeks than when you hit your thumb (with a hammer). I work with companies, which I visit every two weeks, where people can sign up, and then you might see only those who have had problems for two days. This is a completely different situation than when I see someone after about three months; in the latter situation, far more explanation is needed.* (Expert 14)

#### Social support and asking for help

According to the experts, people suffering from CANS for ≥ 12 weeks are in the chronic stage of CANS. Often, they do not know what they can do to reduce their symptoms. One expert indicated that there is a hidden need for reassurance in this group:

*The first non-verbalized need - is that of reassurance. There are many people who say that it will never get better. When repetitive strain injury first appeared 15 years ago, the major newspapers went along with this: if you ever develop it, you will never get better. (…) What I did was to try to say that this is the situation right now, at this moment in time it’s not going very well, but you don’t have rheumatism or any other similar condition.* (Expert 14)

Experts said that in the group sessions people can recognize themselves and their problems and feel supported. Experts found that the exchange of experiences is a particularly important advantage of the group sessions:

*… and then they learn a lot from each other, and see that ‘yes’ you also have this, I also experience it in the same way … and then someone tells how he dealt with it and then the other person thinks – ‘I’m going to try the same thing’. My experience is that discussing something like this takes an enormous amount of time.* (Expert 16)

The experts noted that it is sometimes difficult to properly formulate a request for support, and to discuss experiences and needs with the supervisor at the right moment. Therefore, employees should acquire the tools to communicate with their supervisor. One expert indicated that workers themselves must decide whether and how they want to talk with their supervisor, or perhaps choose another possible solution:

*I believe that people have to think about that on their own - if you personally want to change something in your work environment then you have to consider that you will have to discuss this point. That’s the approach which you have chosen for yourself, because you could choose different solutions which would not involve the need for this discussion.* (Expert 5)

According to some experts, relationships at work may play a role in the onset or persistence of complaints. It is important to reflect on the work environment and relationships:

*… important is the work situation, the employer, how the work is organized and how the different spheres of influence work out. It’s valuable to provide information in recognisable themes, perhaps also use role playing… but make it on a larger scale than only focusing on the employee with arm, neck and shoulder complaints.* (Expert 10)

Experts believed that employees with complaints can feel very unhappy if an employer does not cooperate. Social support was considered very important. Workers should be able to obtain social support from colleagues, managers, friends, family and/or healthcare professionals. One expert stated that support at the workplace, as well as in the private sphere, is one of the most important issues to be addressed. Knowing how to obtain these resources of social support, without feeling threatened, is an important skill. Employees are not always aware that this lack of workplace support may be an extra burden. In addition, there may be psychosocial factors at home, whether temporary or not, affecting the complaints or the personal capacity. Experts considered it important that employees are aware of these possible factors.

Experts mentioned communication as an important topic which can stimulate social support. Good communication starts with self-reflection: How do I communicate? In addition, suggestions for good communication were considered important, including training of communication skills. Employees with CANS are often highly engaged with their job and do not easily say ‘no’. More assertiveness towards the employer may be required. It is also important that employees acquire the tools and skills to communicate with their supervisor, e.g. about their needs and experiences. Employees may also feel that their supervisor does not listen to them, so it is useful to examine how employees communicate their needs and experiences:

*I occasionally meet people who say ‘I want another computer mouse but I don’t think my direct manager allows me to’. Then I ask whether he/she already made this request to his/her boss. That piece of competence - to approach your boss with your request for help - is important.* (Expert 7)

### Behaviour

#### Self-efficacy and empowerment

Experts saw a role for a self-management program for employees with CANS and agreed with each other that the intervention should focus on increasing the employee’s self-efficacy and empowerment. Employees must have the confidence to handle situations they are confronted with in the right way. Participants of a self-management program should also be challenged to take the lead in the management of their complaints. To achieve this, information may be provided, skills can be trained, and participants must identify possible solutions themselves. By offering a wide range of information and knowledge, and by practicing skills, each individual employee can select for themselves the relevant topics and then take action. Regarding the breadth of the information that should be provided, one expert said:

*I would say that it must not only be about the arm, shoulder and neck, but primarily about work, about yourself, and how you manage to restore yourself to a good balance. And starting to work and continue working on a healthy way.* (Expert 10)

#### Taking action

No ready-made solutions should be offered. Participants should be facilitated to find a tailored solution. Participants themselves must take action and find solutions; in this way they will also be highly motivated. Participants must consider various solution options and make choices between them. One expert stated that three possible solutions must be available before one can make a ‘real’ choice. According to experts, an additional eHealth module could have added value because it may provide ideas for possible solutions. It is important that participants consider their own solutions; these will differ for each individual depending on the underlying problem (s) and personal situation. One expert explained:

*I do believe that - which also is the challenge - to let it come from themselves. To use what they experience as support. Each person has his/her own manner.* (Expert 6)

It was assumed that the target group of the intervention, i.e. workers with longer-term CANS, are open to such an approach. Employees with CANS have often taken various steps with the aim to reduce their complaints.

#### Setting goals and making choices

Setting goals was indicated as important. Experts considered it important to split the main goal into sub-goals. Achieving some success in between can also work as a motivating factor. It is also important that participants feel strengthened. People gain confidence as they tackle a part of the problem and gain control over this problem. This increases the chance that, once the program has ended, the participants will continue working in this way.

*I also think it’s very rewarding if you really do have actual complaints and you have learned through reflecting on these complaints, discussing them with people, looking up information on the subject, and by trying out various things - and that you realize that the complaints become less severe over time. I can understand that this approach works well. Also, in different but similar situations, you can perhaps also use the same approach through which you can achieve success.* (Expert 5)

According to experts, another role of a self-management intervention is to ensure that employees are aware of the possible facilities and treatment options (with regard to their complaints) within and outside their organization or company. In this way, employees can more easily find the right facilities and care. Overall, experts believed that participants should be able to make their own choices. One expert stated:

*For one person it mainly concerns the development of talent, identifying your own strengths and then using these optimally. For another person it involves the physiotherapist coming by and then, together with your employer, you determine where you can find the financial resources to obtain a better monitor.* (Expert 15)

#### Important skills and behaviour

Besides communication skills, according to some experts, other important skills can be related to physical activity, private life, load and capacity, setting limits, taking breaks, relaxation and ergonomics. Experts stated that participants must realize that what is good for one person may not be good (or not useful) for another.

##### Physical activity

Some experts in the focus group who had employees with CANS tried to improve their complaints through sports/exercise and tried to upgrade their physical capacities. Experts agreed that the importance of physical activity should be emphasized and participants should be encouraged to undertake more physical activity. Exercises were also considered important. Experts said that physical activity and exercise must be gradually increased, because muscles may not be in optimal condition; in some cases activity should be supervised by a physical therapist. One expert stated:

*One of the causative factors is also the lack of movement, and fear of movement.* (Expert 3)

##### Private life

Experts stated that it is important that employees have sufficient relaxation in their spare time. A good balance between work and home activity was considered important. Concentrating on one’s hobbies and interests can help with this. In addition, the home situation can also be a physically stressful factor, as indicated by one participant:

*A lot of people work at home - many people work on the computer or are gaming online, have painted the ceiling, or have laid paving stones for a sidewalk.* (Expert 15)

##### Load and capacity

Physical capacity can vary greatly from person to person. Employees can influence this by adjusting/lowering the load, or increasing their physical capacity. According to experts, employees should give priority and listen to signals from their own body. Employees should correctly estimate their capacity, set their limits, and ask for help from others when needed. One needs to find a good balance between one’s load and one’s capacity:

*That is therefore the balance: which means that you know from experience that if you don’t set this limit, then you will develop very serious complaints.* (Expert 5)

##### Setting limits

As mentioned, employees must set their own limits; this was considered as an important skill. Experts also indicated that setting limits is not a convenient term in relation to self-management. In fact, in an intervention focused on self-management, participants should find their own solutions.

Experts stated that the experienced problems should therefore give rise to looking for solutions and alternatives. Employees with CANS should realize that if they carry on without changing anything their complaints will worsen, and then alternatives and solutions will also be more difficult to find. Setting their own limits could be a part of this solution. However, this is not always easy, for example in certain occupations:

*I also see this during clean-ups or in the cantina where people have to repeatedly perform the same activities. Then you cannot easily say that you need to take your time. (…) I recognize this situation quite clearly in administrative work. In all work situations there is this constant pressure to keep working at all costs.* (Expert 2)

##### Taking breaks

Taking regular breaks was considered important. Employees need to take a break at certain time intervals and not wait until they experience symptoms. One expert reported that, in some companies, taking a break is obligatory because of the increased risk of developing complaints when persisting with work. Employees are, for example, also encouraged to get up and move around during the breaks. About the role of taking breaks one expert stated:

*There is a logic underlying the link between the development of complaints and the duration of the period when this actually occurs. There are intermediate stages, which precede the actual appearance of the complaints. If someone becomes aware of the fact that he has complaints after one and a-half hours, he could also have become aware of this within three-quarters of an hour when the first complaints became evident, if he’d known how the symptoms would manifest. I think that someone has to take breaks earlier.* (Expert 3)

##### Relaxation

Experts believed it is important that participants receive information about stress and relaxation. Also, information on the negative effects of stress and information on stress in relation to the development of symptoms are considered important. Information on activities in relation to muscle tension is also helpful. Some experts stated that practical advice on how to relax (muscles) is essential.

##### Ergonomics

Information on the ergonomics of the workplace is valuable: e.g. how to adjust the desk, chair and monitor, and the proper use of keyboards and/or mouse. One expert remarked:

*On a completely different level, it’s just about the competence to adjust your office chair.* (Expert 10)

Other aspects such as lighting, sound, climate, working posture, and work techniques were also important topics. Also, the ergonomics of the workplace at home was considered a matter of concern, because many people use a laptop at home where posture is often far from optimal. Within the framework of alternative workplace strategies this topic must also be addressed.

### Combination of self-management sessions and eHealth

Experts indicated that the combination of group sessions and eHealth can work extremely well. The sessions and eHealth can strengthen and complement each other. Topics may be initiated in the sessions and participants can, if interested, sort these out in the eHealth module. Additional assignments or exercises can also be offered in the eHealth module. In general, experts endorsed the additional value of the eHealth: as one expert stated:

*I really do view eHealth as a very definite support to this. (…) In fact, it can be considered as an additive you can offer to the palette … and a great way in which you can provide a lot of information. Through this approach people can very selectively choose what they need.* (Expert 8)

The self-management intervention was seen as a roadmap, in which participants work on their personal goals, and have interaction with other participants. The eHealth module lends itself to provide more information. Participants could then use this information in the sessions in order to achieve their goals. Participants can use the eHealth to solve the formulated problems and fulfil their action plans. Because CANS has a multifactorial origin, experts expected that eHealth can offer the opportunity to sift through a considerable amount of information. The eHealth module is ideally suited to address all dimensions of the related topics. It is important to determine in advance which topics should be addressed in the self-management sessions, and which topics should be covered in the eHealth. Regarding what should be addressed in the meetings and the eHealth one expert stated:

*For example, about office skills and adjustment to the office chair. Perhaps you actually don’t do this in the sessions - but rather (a discussion of) a very distinct office chair and a description of the five most popular office chairs.* (Expert 10)

Generally the experts saw the self-management sessions as the main focus of the intervention, with the support of the eHealth module. The eHealth is thought to contain additional information, including scientific publications. In the eHealth it is also possible to look at topics from another perspective. Experts indicated that it is important to facilitate use of the eHealth module, e.g. by referring to this in the self-management sessions.

According to the experts, the eHealth should be self-explanatory with short and concise information. It should be attractive and could include a forum or an online community. An overview of the experts’ opinions on the conditions and requirements concerning the layout and design of the eHealth module are presented in Table 2. The most important items regarding the intervention as reported by the experts are presented in Table 3.

**Table 2:** **Conditions and requirements concerning the design, layout and interactivity of the eHealth module**

**Design**	The eHealth should be designed in such a way that people can work with it themselves and can search for possible solutions. It should be self-explanatory.*… and with as little distraction as possible. The person has to immediately understand the correct button to be clicked on… and a short demonstration film, that sort of thing, is also often crucial. That its use does not represent a barrier to continue… and indeed, you must not want to fill in a website, no long texts. Visual support as much as possible, then you have to achieve something with a drawing/record or something interactive* (Expert 9)
	There must be a guiding line: for example, phases or themes. Some parts can be obligatory and other parts can be optional.
	The information should be short and concise. With the use of tabs: so that it is possible to distinguish between the main themes and to distinguish several levels.
	If possible, the eHealth should be designed as an independent program, so that in the implementation phase it can be used without the group meetings. In some sub-groups the eHealth itself may give sufficient support.
**Layout**	The layout should be attractive. *Irrespective whether or not people find the concept of eHealth appealing, the way you present it - the interface - its attractiveness is very important.* (Expert 13)
	Paying attention to apparently ‘smaller’ details is important: for example, the font that is used. What seems trivial may have considerable influence.
	Preferably use images, video and/or voice messages.
**Interactivity**	There is some discussion as to whether the website should be interactive. On the one hand this makes the website more attractive, but eHealth then becomes more complicated - which is not desirable for this purpose. These considerations should be evaluated. *If you say interactive then you first have to have a goal to reach - and only then can you say interactive or not.* (Expert 13)
	Implementing a diary feature is a possibility: *Regarding a diary - hopefully most participants won’t have any objection to fill in a diary on the computer. If they already do that, then it’s a good preparation for the next session. If people want to share the diary with each other – then they can.* (Expert 10)
	But another expert stated: *What is of course also interesting, is that there are people with complaints that arise from regular computer use* (Expert 10)
	Therefore, use of the computer for additional features needs to be considered, in order to prevent more hours spent behind the computer.
	Experts have different opinions about adding a forum/community with participants and experts. A community with healthcare providers is frequently used nowadays, and an online consultation is also an option. However, a forum/community has the disadvantage that participants might ‘whine’ about their complaints. Moreover, participants can contact each other in the group meetings and with small groups it is difficult to have an active community online.

**Table 3:** **Most important item as reported by the experts**

**Group 1**	
Expert 1	*On looking back, I think that you look back together with your colleagues. The colleague has done many things that she/he reflects on her/his own activities so that she/he feels stronger or learns from it. You only achieve the effect if you ask to reflect.*
Expert 2	*I think it’s very important that you convey some degree of enthusiasm, so that they become convinced that you yourself to a large extent possess the key to the solution. And that you need some additional help with this - then they can go and do it on their own.*
Expert 3	*I think that knowledge on the symptoms and on the consequences of the symptoms for the activities that these people are carrying out is important information.*
Expert 4	*A little understanding, development of insight into the risk factors, and in this way to be able to work out what to do with it in more detail.*
Expert 5	*I have written down ‘socially desirable behaviour, assertiveness and social skills’.*
**Group 2**	
Expert 6	*One thing that I find important is that people learn to feel what their body is telling them - and learn to listen to their body. And also to once again come into contact with themselves - a little bit of mindfulness.*
Expert 7	*For me it is important that people can establish a connection between what they are doing and the effect of what they are doing on their body. (…) And perhaps quite simple, but to celebrate successes. People sometimes find it quite normal that an action is successful. Subsequent processes are sometimes small steps but ones which are important to someone - for these you certainly require courage, perseverance, insight. Therefore, you may also celebrate the success that you have actually accomplish.*
Expert 8	*I say: the user interface of the eHealth. Therefore, that what people see is attractive.*
Expert 9	*I think what is important is the retention, the retention of the effect of the treatment. That there is a way to prevent relapse.*
Expert 10	*Perhaps the deeper question is what I consider to be more important, the pain in my arm or my work. (…) And about work load and capacity to work, the making of choices, most certainly with those people whom you know will always have minor complaints - they have to set priorities. Then the question which remains is what do I think is the most important.*
Expert 11	*I think I should say work ethics, norms and values. When do you find yourself (to be) a good employee. What are your criteria?*
**Group 3**	
Expert 12	*What I just said - the evaluation process, and what I said in the beginning - safety and support. That is very important.*
Expert 13	*I would in any case include physical activity, the stimulation of more physical activity in the program.*
Expert 14	*I think, whether you focus on the work environment or on the physical aspect, in both cases cognition is essential. How do people personally think about these things. There are many incorrect prejudices and opinions. Often there is too little knowledge about the human body.*
Expert 15	*What we just said - that people acquire insight into how behavioural changes work and how do I personally view such changes. In which phase are you - and how is that going - so that they also understand why their goals are not being reached. That you then - once again - can do something. Insight into behavioural change is very important.*
Expert 16	*The role of the supervisor in the development and solving of the problem.*
Expert 17	*I think that you also really do have to support the use of the website.*

## Discussion

Experts seem to see a role for a self-management program for employees with CANS. However, as shown in Table 3, many items are indicated to be important by the experts. Experts emphasized that an intervention that aims at understanding or, moreover, decreasing CANS in employees, should focus on increasing employees’ self-efficacy and empowerment. Employees with CANS have difficulty in managing their own health problem and their work. Informed awareness and behavioural change are considered important for this group of employees. Complaints will not always go away, but a self-management program can offer support to these employees.

Experts indicated that self-management begins with the intention to take action. Self-management starts from awareness of a personal problem of the employee. It is very important that people come up with their own solutions, are in control, and feel empowered. Providing knowledge can also be a part of a self-management intervention. It can consist partly of creating awareness with regard to possible risk factors, cues to prompt people to become aware, and about how participants can influence these risk factors themselves. According to the experts, self-management also involves self-efficacy; people must develop confidence that they can handle situations that they are confronted with in an appropriate way. The view of the experts on self-management finds support in literature [20,39,40].

Experts indicated that the combination of group sessions and eHealth can work extremely well. The sessions and eHealth can strengthen and complement each other. To our knowledge, no group-based self-management intervention including eHealth currently exists. In a systematic review on the use of information technology for diabetes self-management, no single intervention combined group sessions with eHealth [41]. Topics may be initiated in the sessions and participants can, if interested, sort this out in the eHealth module. In general, experts endorse the additional value of an eHealth module. It is important to determine in advance which topics should be addressed in the self-management sessions and which can be covered in the eHealth module.

During data analyses, it appeared that the identified main categories emerging from the data, showed similarities with the I-Change model (2.0) Therefore, the main categories emerging from the data were clustered according to the three phases (Awareness, Motivation and Behaviour) of the I-Change model [38]. The I-Change model also assumes that behaviour is the result of intentions and abilities and explicitly makes a distinction between three phases of motivational change and their corresponding determinants [38]. In the pre-motivational phase (Awareness), people need to become aware of their risk behaviour. In the motivational phase (Motivation), people need to become motivated to change their behaviour; in this phase, an intention is formed. In the post-motivational phase (Behaviour) people need to translate intentions into actions, so several preparatory actions to facilitate the actual behaviours need to be planned and executed [38]. The I-Change model is built on the Attitude – Social influence – Efficacy (ASE) Model [42] (comparable to the theory of planned behaviour [43-45]), on which the original intervention of Detaille et al. [32,33] was based, and has incorporated ideas from several social cognitive models [38]. As can be seen in Figure 1, the I-Change model assumes that motivational factors are determined by various factors, such as awareness factors, preceding factors and information factors [38]. By using the I-Change model we were able to relate the outcomes of this study to the stages of behavioural change.

In general, experts found it important that the intervention deals with the possible (multifactorial) risk factors related to the complaints and the underlying problems; this is because employees with CANS are often unaware of the diversity of the possible risk factors. Moreover, earlier focus groups with employees revealed that not all employees are aware of the actual cause of their complaints [17]. The multifactorial risk factors of CANS are supported in the literature [4-7,46]. The earlier focus groups with employees with CANS also indicated that basic information about the complaints, including potential risk factors, is needed [17]. The importance of other topics identified in the focus groups with employees [17], such as information on symptoms (including chronic pain), as well as workload and physical capacity, are also endorsed. According to the experts, employees with CANS should be more proactive. Also, in the intervention, difficulties should be identified and participants should make their own choices and obtain reassurance.

Employees with CANS find it difficult to deal with their complaints and may have difficulty in managing prolonged work activities and paying sufficient attention to their physical posture [17]. Dealing with and acceptance of complaints are topics that also arise in relation to other chronic musculoskeletal disorders, such as low back pain [47]. Finding a balance between all the requirements related to activities at work is challenging; therefore, information about the work environment related to CANS, including workplace adjustments, is required [17]. Experts indicated several areas related to the work environment, including workplace ergonomics, that should be addressed in the intervention; therefore, the work environment seems to be an important topic, especially in the eHealth.

The importance of exercises is generally recognized by employees with CANS [17] and is also indicated by patients with low back pain as a way to manage complaints [47]. Some experts recommended that employees with CANS might improve their complaints through sports/exercise and should upgrade their physical capacities. On the other hand, in our focus groups with employees, some participants stopped stressful sports activities because they thought these activities would aggravate their complaints [17]. Experts recognized the value of physical activity and the importance of exercises. Both experts and employees with CANS also indicated the importance of having information on and exercises about (muscle) relaxation [17], which is supported by others [48].

Addressing the negative effects of stress, and information about stress in relation to CANS, is considered important by experts. Employees do not always find it easy to deal with the stress and pressure of work [17]. Experts stated that employees must set their own limits and that this is an important skill. Related to the setting of limits, employees with CANS have a relatively high threshold before asking for help, whereas others think they should tighten up their limits [17]. Focus groups with employees identified a relationship with stress in the development and worsening of their complaints [17]. In fact, work stress is associated with common health complaints, such as musculoskeletal pain [49]. Moreover, (work) stress is associated with musculoskeletal problems of the upper extremity [50]. Also, employees with CANS indicated that taking into account one’s own limits is important [17].

Experts considered communication skills to be important. Employees with CANS did not always find it easy to talk about their complaints and/or to bother others about their problems [17]. Generally, there are no major problems encountered with communication, but employees with CANS considered providing communication tools for discussion with others about CANS to be important [17].

Social support is considered valuable by the experts. Patients with low back pain considered emotional support and encouragement as essential [47], and social support was also considered important by patients with rheumatoid arthritis [51], which emphasizes the importance of this topic. In general, employees with CANS experienced sufficient support from their colleagues and from those at home [17]. Most employees experienced sufficient support from their supervisor; however, some employees who participated in earlier focus groups experienced insufficient or no support from the supervisor [17]. This could indicate that knowing how to obtain social support is also an important skill.

Experts found it important to address the importance of finding a good balance between work and home. This is endorsed by some participants in the focus group with employees complaining that there is a lack of balance between their work and private life [17]; this can also occur in other chronic conditions, for example a neuromuscular disease [52].

### Limitations of the study

This study has several limitations. First, a total of 17 experts in the field of CANS, self-management and/or eHealth participated; this is probably a rather arbitrary selection of all experts on these topics in the Netherlands. We decided to divide the different areas of expertise into the three focus groups, thereby ensuring that in all focus groups all topics could be discussed. The alternative, i.e. placing all experts of one area together in one group, might have produced more discussion about each of the topics - but separately. By having mixed focus groups all experts participated in the discussions on all topics, which made it possible to establish the relationships between the topics. Although we did not set a point of saturation in advance, it is highly likely that saturation was reached because the same issues were identified and discussed in all three focus groups. Data were coded by one researcher. Multiple coding involves the cross-checking of coding strategies and the interpretation of data by independent researchers [53]. However, as Barbour [53] stated, the degree of concordance between researchers is not very important; the main value of multiple coding is to supply alternative interpretations [53]. It is important that a transparent and systematic process is followed which can be carried out by one researcher, by a team, or by involving independent experts [53]. By discussing the emerging main categories and looking for alternative interpretations for our findings in a small research group, we investigated the potentially competing explanations.

As mentioned in the Introduction, for the specific group of participants with CANS, more prolonged computer use (due to following an eHealth program) could worsen their physical problems. However, this was not specifically mentioned by the experts.

### Content of the intervention

Important topics of the intervention indicated by experts are the possible causes of complaints, addressing potential symptoms, identifying difficulties and problems, making choices, and reassurance and self-awareness. The intervention should also address behaviour such as setting limits, taking breaks and ensuring sufficient relaxation. Ergonomics, social relationships and social support, the importance of physical activity and exercises, and a good balance between work and home activity are also considered important. The topics identified in this focus group study generally meet the needs of employees with CANS [17] which are related to exercises, muscle relaxation, working with pain, work environment, social environment and personal factors (including work style), all of which are supported by earlier studies [6,7,18,19,54-63].

## Conclusions

The present study provides valuable insight into experts’ opinion on a self-management program for employees with CANS. Experts seem to see a role for a self-management program for employees with CANS and the intervention should focus on increasing the employee’s self-efficacy and empowerment. Experts indicated that the combination of group sessions and an eHealth module can work extremely well. Moreover, experts from different fields provided valuable information regarding the development of a self-management program for employees with CANS, which can be used in the adaptation of a self-management program following the intervention mapping protocol [35]. This information can also be used to develop other interventions and for the treatment of employees with CANS.

## Appendix: Interview guide for the focus group sessions

### Introduction (5 min)

- Brief introduction about the research project and the aim of the focus group.

### Introductory questions (20 min)

- Introduction of the participants (name, type of work, experience with CANS, self-management and eHealth).
- What is self-management for employees suffering from CANS?
- Does a self-management program for employees suffering from CANS for more than 12 weeks make sense?

### Content of the intervention (55 min)

- Which topics should be addressed in a self-management intervention for employees with CANS?
- What kind of information about the onset and persistence of CANS needs to be discussed in the intervention?
- What kind of information about dealing with and reducing complaints should be included in the intervention?
- What kind of skills should employees with CANS have developed after the intervention?

### Development of the intervention (30 min)

- Which requirements should a good eHealth module fulfill for the target group?
- What are potential pitfalls and/or important points in the development of an eHealth module?
- Which requirements should a good self-management program for the target group fulfill?
- What are effective methods to use in the self-management program?

### Self-management program including eHealth (20 min)

- Which topics, or what kind of topics, should be covered in the self-management sessions and which topics, or what kind of topics, should be addressed in the eHealth?
- How can the self-management program and eHealth module complement each other?

### Closure (10 min)

- What would you say is the most important topic or item in the goal or the development of the intervention?
- Which topics did you miss during this meeting, but are important in the process of developing the intervention?

## Abbreviations

CANS: Complaints of the arm, neck and/or shoulder
WMO: Dutch law on ‘Medical Research involving Human Subjects’
